# Hypothermia: effects on platelet function and hemostasis

**DOI:** 10.1186/s12959-014-0031-z

**Published:** 2014-12-09

**Authors:** Sven Van Poucke, Kris Stevens, Abraham Emanuel Marcus, Marcus Lancé

**Affiliations:** Department of Anesthesiology, Intensive Care Medicine, Emergency Care and Pain Therapy ZOL, Genk, Belgium; Department of Anesthesiology, Maastricht University, Maastricht, Netherlands

**Keywords:** Platelets, Hypothermia, Coagulation, Hemostasis

## Abstract

Mild therapeutic hypothermia is considered standard care in the treatment of patients resuscitated from cardiac arrest. With increasingly more frequent concomitant use of platelet-inhibiting drugs, clinicians must be cognizant of the ramifications of hypothermia on platelet function as part of hemostasis. The effects of hypothermia on platelet function have been studied for more than 50 years, but the results are inconsistent and may be related to the circumstances during which hypothermia is achieved. This review summarizes current knowledge of platelet function during hypothermia and the impact on hemostasis.

## Introduction

Although humans are homeothermic, significant body-temperature changes can result in life-threatening situations (including bleeding and thrombosis), particularly in association with certain medical conditions [1-10].

Platelets lack nuclei, but perform multiple vital functions of nucleated cells. Platelets can generate new cell bodies packed with respiring mitochondria and α-granules [11]. Whereas platelets were once considered to function exclusively during hemostasis and thrombosis, they are now considered to function as circulating sentinels in the activation, modulation of the host immune response [12-14].

This review summarizes current knowledge of platelet function during hemostasis under various hypothermic conditions. The review will not discuss the effect of systemic hypothermia on the pharmacokinetics and pharmacodynamics of anti-platelet drugs [15-19].

## Review

### Fundamental aspects of hypothermia

The research on hemostasis in the setting of hypothermic reveals inconsistent, even conflicting results, ranging from seasonal increase in thromboembolic disease in winter to excessive surgical bleeding in hypothermic patients.

The effects of hypothermia [20] on coagulation and platelet function is influenced by

- the actual body temperature during sampling
- the pre-analytical and analytical temperature and sample type (in-vivo, ex-vivo, in-vitro; whole blood, washed platelet preparation)
- temperature changes during the sampling time (induction, maintenance, and rewarming)
- the moment of sampling in relation to agonist stimulation
- the duration of hypothermia
- the cause of hypothermia (spontaneous, whether induced externally or internally)
- coexisting factors (extracorporeal circulation [21], comorbidity, drugs)
- the modality of induced hypothermia (local, regional, or general) [2]

Hypothermia can be caused by metabolic dysfunction in association with decreased heat production (hypothyroidism, hypoglycemia, or hypoadrenalism) or disturbed thermoregulation (intracranial tumor or degenerative neurological disorders). Accidental hypothermia is an unintentional decrease of core temperature caused by prolonged exposure to cold [22]. Hypothermia acts as a natural survival strategy in some animals that hibernate, and actively suppresses metabolism. [4,23,24].

In trauma patients, extra precautions are required based on inherent bias due to absolute or relative hypovolemia and acidosis [25].

Since the early 1950s, active therapeutic cooling has been used during specific surgical procedures to reduce oxygen requirements of organs such as the brain, heart, and kidney [26,27]. The use of hypothermia has recently been extended to post-resuscitation care based on results from more intensive and innovative monitoring techniques [28-31]. While precluding the current guidelines for platelet storage, research on chilled platelets (at 4°C, ex-vivo) should be differentiated from research on deep hypothermic circulatory arrest at in-vivo temperatures of 15-18°C and from research on cardiopulmonary bypass and post-resuscitation at temperatures > =28°C.

Hypothermia has been shown to result in hemoconcentration, leukopenia and thrombocytopenia, slowing down of coagulation enzymes, disordered fibrinolysis, and disruption of platelet function [6,32-34]. Some hematologic diseases are directly influenced by temperature changes; for example, cold agglutination disease exhibits an increase in cold agglutinin titers [35].

With more frequent use of hypothermia in clinical practice and concomitant use of platelet-inhibiting drugs, there is a growing need to understand the ramifications of platelet-inhibiting drugs on coagulation and platelet function [36,37].

### Effect of hypothermia on platelets

#### Platelet morphology

Chilling platelets (4°C) *in vitro* results in volume increase, spherical deformation, and the formation of lose marginal microtubules and pseudopods [38,39]. The chilling-induced (0°C, ice water), reversible shape-change in platelets correlates with phosphorylation of myosin, subsequent interaction on actin filaments and free cytosolic calcium increase [40]. Human platelets can be maintained in a discoid shape in the cold, *in vitro,* using a cell-permeable calcium chelator to attenuate calcium mobilization and cytochalasin B to prevent barbed-end actin assembly [39]. FTIR spectroscopy in northern elephant seals confirms three different thermotropic membrane phase transitions [1]. The microtubules of hibernating mammals are more tolerant to cold, which facilitates the rapid shift from a thrombocytopenic, anticoagulant state during torpor to a normal state [3,4].

#### Platelet function

Platelet adhesion and activation leads to their aggregation and ultimately to the formation of a fibrin-rich hemostatic plug [41]. Hypothermia promotes platelet margination by increasing hematocrit, changing platelet shape, lowering blood flow rate, and increasing the expression of adhesion molecules.

##### Low temperature enhances shear-induced platelet aggregation

Platelets interacting with the vessel wall are influenced by the small-scale motions of neighboring erythrocytes, which allows platelets to move across flow streamlines in a form of enhanced diffusion. Platelets contact each other via collisions driven by blood-flow velocity gradients [42]. Adhesive interactions between platelets and the extracellular matrix are strongly influenced by local rheological conditions. Blood is considered a two-phase liquid with a solid–liquid suspension. The viscosity of a liquid is temperature-dependent, and blood viscosity increases with decreasing temperature [43]. Thus low temperature may enhance shear-induced platelet aggregation by increasing blood viscosity [44].

##### Hypothermia and life span of platelets

Chilled platelets subjected to refrigeration before transfusion rapidly leave circulation. Therefore, blood banks store platelets at room temperature [32,45]. The normal *in vivo* lifespan of platelets (7–10 days) does not appear to be affected by hypothermia. A surface-induced deep hypothermia study (20°C) on dogs reported that the mean survival of platelets (4.9 days) is slightly but significantly longer in the hypothermic group compared to that in the control group (4.2 days) [46].

##### Storage, clearance and release underlies the (reversible) thrombocytopenia

The decrease in platelet count observed *in vivo* during hypothermia is reversible as normal body temperature is restored. This change in platelet count is explained by hepatic and splenic sequestration, and possibly margination of platelets, relative to hypothermic depth and duration, and with a maximum decline between 25-30°C [47-49]. Under mild hypothermia, the reduction in platelet count is modest and remains within the normal range [45]. As core body temperature drops below 37°C, platelets become more susceptible to activation by thrombotic stimuli, a phenomenon known as priming. Therefore, platelets can act as thermosensors. The ability for priming at peripheral body sites, where temperatures are lower and chances for trauma higher evolved as a protective effect against bleeding, whereas more central body sites (brain and coronary vessels) are more protected against thrombosis [39].

Subjecting platelets to chilling changes its surface configuration. In response to cooling, the GPIbα subunit of the vWf receptor complex undergoes clustering and becomes a target for recognition by hepatic macrophage complement receptor type 3 (CR3), which is strongly expressed in liver macrophages, and leads to platelet phagocytosis and clearance. Compared to mice that are CR3-deficient, mice overexpressing CR3 demonstrate a rapid reduction in platelets counts when exposed to cold leading to platelet phagocytosis and clearance in the liver [47].

##### vWF retention is prolonged on the cell surface at low temperatures

vWF is a protein that circulates in a globular form under conditions of low shear-stress, but changes into an elongated form under the influence of stronger hydrodynamic shear forces [50,51]. Expression of vWF in endothelial cells is higher at low temperature than at normal temperature [52]. The kinetics of vWF proteolysis by the cleaving metalloprotease ADAMTS-13 is temperature-dependent, with slower but complete activity at 4°C and at 22°C. A sub-physiological temperature might influence the proteolysis kinetics due to minor variations in ADAMTS-13 structure, or further modification of the vWF substrate [53]. The failure of secreted vWF to form long cell-surface strings following its secretion at low temperatures (≤17°C) results in formation of predominantly globular deposits. This failure of vWF to unfurl at lower temperatures, combined with its reduced thermal motions, may interfere with the prolonged retention of this protein on the cell surface, and may result in hemostatic disorders [54].

##### The recognition of vWF with factor VIII is sensitive to temperature changes

Closely related to vWF is factor VIII which, after its extracellular release, forms a complex with vWF [51]. Thermodynamic analysis reveals that the recognition process of factor VIII with vWF is very sensitive to temperature changes. Generally, interactions between proteins with pre-optimized binding sites are stimulated by increases in the system kinetic energy (temperature). By contrast, interactions between proteins driven by conformational changes are generally reduced by temperature increases. The stimulatory effect of higher temperatures on the association kinetics and affinity of factor VIII for vWF suggests that this interaction does not require significant conformational changes [55]. The impact of body temperature changes on the recognition process of factor VIII with vWF is currently unknown.

##### Hypothermia increases the ability of platelets to respond to activating stimuli

Moderate hypothermia results in a minor increase in spontaneous platelet activation but a significant rise in agonist- induced responsiveness. In-vitro research in mice demonstrates that with incubation at temperatures of 34°C and 31°C, spontaneous expression of P-selectin and the activated conformation of GPIIb-IIIa does not change markedly. A small yet statistically significant increase in PAC-1 binding in unstimulated samples at 31°C suggested spontaneous hypothermia-induced activation. TRAP exposure during hypothermia causes an increase of PAC-1 binding with increased activation during hypothermia. In line with this, binding of fluorescent-labeled fibrinogen increases at 34°C and 31°C after TRAP exposure [56].

Although early research only referred to cold effects on platelets as “activation,” chilled platelets do not resemble platelets activated by classical agonists such as thrombin, ADP, or collagen. Whole blood aggregation assays demonstrated that platelet aggregation and P-selectin expression are enhanced under hypothermic temperatures but the effect depends on the agonist used. The potency of the agonist does not seem to be related to the susceptibility of platelets to the effects of temperature [57-68].

Interestingly, the platelet intrinsic function is maintained throughout torpor/arousal in hibernators as well as throughout cooling/rewarming and pharmacological induced torpor, as demonstrated by P-selectin expression and platelet aggregometry. P-selectin expression on circulating platelets, however, are significantly decreased in torpid hamsters, but restores to normal euthermic levels shortly after arousal [49].

##### Factors regulating thrombus formation may be tissue and temperature dependent

Mechanisms that limit or prevent the process of thrombus growth are essential in the balance between prothrombotic and antithrombotic forces. Cold-induced vasodilatation mediates cyclic regulation of blood flow during prolonged cooling of protruding limbs, reducing localized cold injury [69]. The protective effect of NO and prostacyclin on platelet aggregation during temperature-dependent vasoconstriction and vasodilation, is currently unknown but might be tissue and temperature dependent [70].

##### The machinery for executing platelet apoptosis is temperature dependent

Studies on the effects of chemotherapeutic drugs revealed that apoptosis in platelets, as determined by mitochondrial inner membrane potential depolarization is much more efficient at 37°C than at room temperature [71]. Cold-storage of platelets followed by rewarming has been shown to trigger apoptosis through a GN-sensitive GPIbα-change indicative of receptor clustering [72].

## Conclusion

The impact of hypothermia on platelet function and its effect on hemostasis has been studied for more than 50 years, yet its effects and the mechanisms behind the observed phenomena have not been fully elucidated. Studies differ in the circumstances under which hypothermia is achieved, and in the duration and extent of temperature decrease. Comparative studies are challenging as the parameters defining sufficient platelet function have not been clearly identified, and experimental studies have not used standardized techniques and platelet-stimulating agents. Conflicting results suggest that the heterogeneous techniques do not accurately reflect *in vivo* hemostatic function, which involves platelets, coagulation factors, plasma proteins, endothelial and other cells and flow characteristics. Animal models may not be directly translatable to humans as demonstrated in hibernating mammals. The hypothermia-associated coagulopathy is more likely related to a reduced availability of platelet activators, rather than a consequence of an intrinsic defect in platelet function. More research is required to elucidate the activation of platelets, the interaction of platelets and leukocytes and the production of proinflammatory cytokines at different temperatures are required.

## Abbreviations

°: Degrees
C: Celsius
F: Fahrenheit
L: Liter
kDa: kilodalton
GP: Glycoprotein
vWF: von Willebrand factor
α: alpha
β: beta
FTIR: Fourier transform infrared spectroscopy
CR3: Complement receptor type 3
ADAMTS: A disintegrin and metalloproteinase with thrombospondin motifs
TRAP: Thrombin receptor activating peptide
ADP: Adenosine diphosphate
FeCl_3_: Ferric chloride
CPA: Cone and platelet analyzer
NO: Nitric oxide
PAF: Platelet-activating factor
BH: Bcl-2 homology domain
PT: Prothrombin time
APTT: Activated partial thromboplastin time
ACT: Activated clotting time
TEG: Thromboelastography
ROTEM: Rotational thromboelastometry
MA: Maximal amplitude
MCF: Maximum clot firmness
PFA: Platelet function analysis
PAC-1: Monoclonal antibody against GP IIb-IIIa
